# Endoscopic recanalization of a completely obstructed colorectal anastomosis using magnets

**DOI:** 10.1055/a-2325-2464

**Published:** 2024-06-05

**Authors:** Willian F. Igi, Isabela Andrina Ribeiro da Silva

**Affiliations:** 1 Endoscopy Unit, Hospital de Amor da Amazônia, Porto Velho, Brazil; 2 Endoscopy Unit, Advanced Digestive Endoscopy Center of Rondônia, Porto Velho, Brazil

A male patient who had undergone emergency loop colostomy because of intestinal obstruction secondary to a sigmoid colon tumor subsequently underwent elective rectosigmoidectomy. Endoscopy prior to bowel reconstruction revealed complete stenosis of the colorectal anastomosis ( Fig. 1 ). A previous attempt at recanalization using a needle-knife was aborted due to perforation. Following a multidisciplinary discussion, the decision was made to perform magnetic compression anastomosis.

**Fig. 1:**
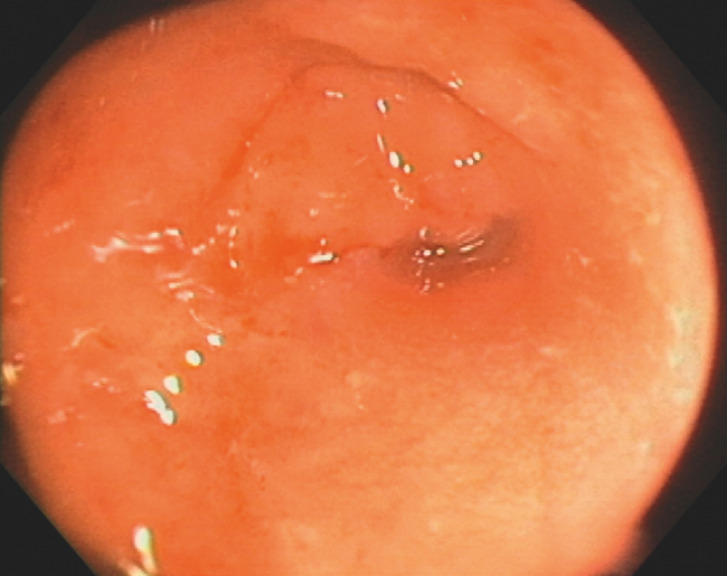
Endoscopic view of oral aspect of completely obstructed colorectal anastomosis, following emergency loop colostomy and subsequent rectsigmoidectomy.

Two 10 × 5-mm neodymium magnets were inserted endoscopically through the efferent loop and rectum and positioned on the oral and rectal sides of the completely obstructed anastomosis. After 4 days, a follow-up colonoscopy revealed recanalization of the stenosis ( Fig. 2 , Video 1 ). Additionally, balloon dilation using a 15-mm-diameter hydrostatic balloon was performed to achieve an optimal caliber. The patient underwent bowel transit reconstruction after 6 months and remained asymptomatic at the 1-year follow up.

**Fig. 2:**
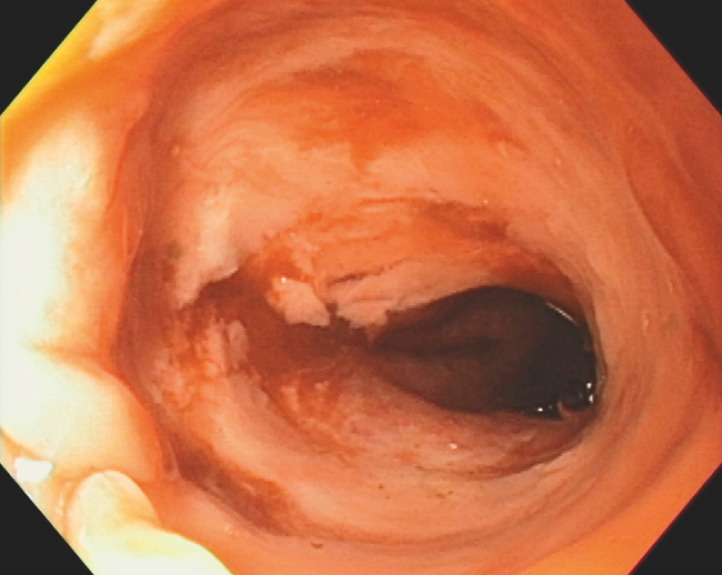
Colonoscopic appearance of the colorectal anastomosis recanalized using magnetic compression.

Endoscopic recanalization using two magnets positioned on the oral and rectal sides of the obstructed anastomosis in a male patient with intestinal obstruction secondary to a sigmoid colon tumor.Video 1

Magnetic compression anastomosis is widely performed in the biliary tract and esophagus but is still in the developmental stage for the gastrointestinal segment. A previous case series indicated its safety and effectiveness 1 . Case reports have described colorectal anastomosis recanalization through techniques such as stricturotomy 2 or in combination with endoscopic ultrasound 3 . Nevertheless, when access to both sides of the obstructed anastomosis is possible, the use of magnets emerges as a technically easier alternative.

Endoscopy_UCTN_Code_TTT_1AQ_2AF

## Notes

Endoscopy E-Videos https://eref.thieme.de/e-videos
*E-Videos* is an open access online section of the journal *Endoscopy* , reporting on interesting cases and new techniques in gastroenterological endoscopy. All papers include a high-quality video and are published with a Creative Commons CC-BY license. Endoscopy E-Videos qualify for HINARI discounts and waivers and eligibility is automatically checked during the submission process. We grant 100% waivers to articles whose corresponding authors are based in Group A countries and 50% waivers to those who are based in Group B countries as classified by Research4Life (see: https://www.research4life.org/access/eligibility/ ). This section has its own submission website at https://mc.manuscriptcentral.com/e-videos .
